# Succession Dominates Alpha Male Replacement in Despotic Rhesus Monkeys: Insights from a Long-Term Study in the Taihang Mountains, Henan Province, China

**DOI:** 10.3390/ani16101495

**Published:** 2026-05-13

**Authors:** Haotian Xu, Bo Zhi, Longhui Hu, Jundong Tian, Jiqi Lu

**Affiliations:** 1 School of Life Sciences, Zhengzhou University, Zhengzhou 450001, China; 16638114660@163.com (H.X.); 18568637709@163.com (B.Z.); 15890956623@163.com (L.H.);; 2 Institute of Biodiversity and Ecology, Zhengzhou University, Zhengzhou 450001, China

**Keywords:** alpha male replacement, rhesus monkey, succession, rank reversal, group fission

## Abstract

Alpha male replacement occurs in many group-living non-human primates, but its characteristics remain under-explored. Species of the genus *Macaca* live in multi-male, multi-female groups and are structured into four levels of social styles, which may impact alpha male replacement. Rhesus monkeys (*Macaca mulatta*) are classified as Grade 1, yet little is known about alpha male replacements in this species. This study investigated the occurrence and characteristics of alpha male replacements in rhesus monkeys inhabiting the southern end of Taihang Mountains, China. The types of alpha male replacements included succession (8/11), Rank Reversal (1/11), and Group Fission (2/11). The average age at which adult males took the alpha rank was 10.2 (±4.1, *n* = 11) years of age. Their average social rank prior to attaining alpha rank was 3.91 (±3.05, *n* = 11). The average tenure of alpha males after 2017 was 2.6 (±1.4, *n* = 5) years, which appeared shorter than that before 2017 (>7.5 ± 2.9 years, *n* = 4). The occurrence of alpha male replacements did not significantly correlate with group sizes, natality, the ratio of adult males to adult females, or the proportion of immatures. Compared with tolerant *Macaca* species, alpha male replacement in rhesus monkeys tend to be biased toward Succession, a pattern that may be linked to their extremely despotic social style. This study suggests that social style could interact with changes in social structure, deepening our understanding of the evolution of primate social systems.

## Simple Summary

How the social structure of group-living wildlife is organized and changes over time is a key topic in behavioral ecology and sociobiology. In species that form multi-male and multi-female groups, dominance hierarchies are hypothesized to facilitate efficient resource allocation, with alpha individuals often assumed to have priority access to various resources. However, the occurrences and characteristics of alpha male replacements remain under-explored in wildlife due to their unpredictability, especially in long lifespan species. In this study, we collected and analyzed evidence of alpha male replacement in rhesus monkeys (*Macaca mulatta*). Succession accounted for the majority of these replacement events. This finding may be linked to the species’ extremely despotic social style, suggesting an interaction between social style and the evolution of social systems in non-human primates.

## 1. Introduction

In natural environments, resources essential for survival (e.g., food, space) and reproduction (e.g., mating opportunities) are limited for social-living mammals. This scarcity often leads to ritualized or physical competition among adult individuals within a group, resulting in the formation of a linear dominance hierarchy among adults [1,2,3]. In non-human primates, male-biased dominance, which is common in polygynous, dimorphic, terrestrial, and group-living species, has been more extensively studied than female-biased dominance, which primarily occurs in monogamous, sexually monomorphic, and arboreal species [4]. Theoretically, higher social rank grants an individual, particularly the highest-rank individual (i.e., alpha male or alpha female), greater access to scarce resources. However, an alpha individual cannot maintain its position indefinitely due to factors such as aging, diseases, injuries, or challenges from subordinate or extra-group individuals. The event in which an alpha individual is replaced by another one is defined as alpha male/female replacement [4,5]. Although alpha male replacement has been more widely investigated than alpha female replacement in non-human primates, the characteristics of alpha male replacements remain under-explored [4,5].

Based on the origin of the new alpha male (whether from within or outside the group), alpha male replacements in non-human primates have been classified into five types [4,6]. First, Takeover refers to an extra-group male obtaining the alpha position through aggression, as recorded in crested macaques (*Macaca nigra*) [7] and Cayo Santiago rhesus monkeys (*M. mulatta*) [8]. Second, Rank Reversal involves a subordinate co-resident male achieving the alpha position via an aggressive challenge to the current alpha male, as documented in tufted capuchin (*Cebus apella nigritus*) [9]. Third, Succession occurs when a subordinate co-resident male attains the alpha position following the death or disappearance of the group’s current alpha male, as observed in mandrills (*Mandrillus sphinx*) and red howlers (*Alouatta seniculus*) [10,11]. Fourth, Waltz-in describes an extra-group male becoming the alpha male by immigrating into an all-female group, as reported in saki monkeys (*Pithecia aequatorialis*) [12], Thomas’s langurs (*Presbytis thomasi*) [13], marmosets (*Callithrix flaviceps*) [14], white-throated capuchins (*C. capucinus*) [15], and Lar gibbons (*Hylobates lar*) [16]. Finally, Group Fission is defined as a new alpha male emerging from a group split, as documented in baboons (*Papio* spp.) [17,18]. Moreover, a single species may exhibit multiple types of alpha male replacement [5,19]. For instances, the types of alpha male replacement include Takeover (5/9) and Succession (4/9) in crested macaques [7]; Rank Reversal (5/20), Takeover (14/20), and Waltz-in (1/20) in Lar gibbons; and Succession (4/6), Rank Reversal (1/6), and Takeover (1/6) in siamangs (*Symphalangus syndactylus*) [19].

The determinants of alpha male replacement can vary depending on the group composition and the typical dispersal patterns of a given non-human primate species [5,19,20]. For example, when the group size exceeds the typical range, Formosan macaques (*M. cyclopis*) may split into two or more smaller groups, with the newly formed groups subsequently generating their own alpha males [21,22]. In one-male, multi-female species (such as *Rhinopithecus roxellana* and *Papio hamadryas*), alpha or resident male replacement in one-male units (OMUs) involves adult males from other OMUs or all-male units (AMUs), or newly formed OMUs recruiting adult males from outside [23,24,25,26]. In such species, social affiliation between the alpha male and adult females weakens during the three months prior to replacement, and replacement frequently occurs in newly formed OMUs or those with a resident male of ten years [24,26]. In multi-male and multi-female species, alpha male replacement may be carried out by either resident or extra-group males. However, relatively little data are available on the characteristics of such replacements, such as which males attain the alpha position and their basic information [5].

Macaques (genus *Macaca*), belonging to the family Cercopithecidae, comprise 25 species [27,28] and are primarily distributed across South and Southeast Asia [29]. Macaque society is characterized by multi-male and multi-female groups, within which adults form dominance hierarchies [29]. The highest rank in a macaque group is termed the alpha rank, typically held by an adult male, who may be replaced by another adult male from either within or outside the group [5,29]. Based on social traits such as hierarchical steepness and counter-aggression, the social styles of macaques are classified into four grades, ranging from extremely despotic (Grade 1) to extremely tolerant (Grade 4) [30]. Given that social style is associated with distinct patterns of aggression (uni- or bi-directional) and responses to aggression (high- or low- intensity) [30], the types of alpha male replacement may also vary among macaque species. For instance, all alpha male replacements in crested macaques (Grade 4) have involved outside rather than resident males [7], whereas Succession rather than Takeover has been considered the typical pattern in Cayo Santiago rhesus monkeys (Grade 1) [8]. However, solid data supporting Succession-based replacement in Cayo Santiago rhesus monkeys remain lacking, not to mention studies from other populations [8,31,32].

Rhesus monkeys are the most geographically widely distributed non-human primate species, ranging from eastern Afghanistan and western India to eastern China and northern Vietnam [29,33]. Their habitat spans tropical to temperate regions, including semidesert, evergreen and deciduous forests, grasslands, mountainous areas, and rural and urban environments [33]. As omnivores, their diet includes leaves, grasses, fruits, bark, buds, invertebrates, and small vertebrates [33]. Adults exhibit pronounced sexual dimorphism, with adult males being larger (531.8 mm) and heavier (7.70 kg) than adult females (468.8 mm and 5.34 kg) [33]. This species shares the typical social characteristics of the genus *Macaca*, including multi-male and multi-female group composition, female philopatry and male dispersal [29]. Adult individuals within a rhesus group form a strict linear dominance hierarchy, which also extends across matrilines [29,34]. The social style of rhesus monkeys is classified as Grade 1, characterized by extreme despotism and high-intensity aggression [30]. The alpha position is typically occupied by an adult male, who has priority access to resources, particularly those that are limited [8,29]. Given the benefits associated with alpha rank, both resident and outside males may compete for the alpha position within a group. However, evidence regarding alpha male replacement in rhesus monkeys remain scarce [8,31,32], particularly concerning the characteristics of alpha males and the alpha male replacement events themselves [5]. From 2013 to 2025, we recorded eleven cases of alpha male replacement in rhesus monkeys natively inhabiting the southern end of Taihang Mountains, China. We therefore predicted that the types of alpha male replacement in this species would mainly involve Succession, consistent with its extremely despotic social style. Additionally, we investigated potential factors associated with alpha male replacement.

## 2. Materials and Methods

### 2.1. Study Area

The study area was located in the Taihangshan Macaque National Nature Reserve (Jiyuan section) (34°54′–35°42′ N, 112°02′–113°45′ E), Henan Province, China. Situated at the southern end of the Taihang Mountains, the reserve spans the mountainous regions of Jiyuan, Jiaozuo and Xinxiang cities, Henan Province, China. The reserve has a narrow north–south dimension (maximum: approximately 15 km) but is elongated in the east–west direction (maximum: approximately 150 km). Its geological features are characterized by steep escarpment and deep valleys, with an average elevation of approximately 800 m above sea level [35]. The southern end of Taihang Mountains experiences a warm temperate continental monsoon climate, featuring hot, humid summers and cold, dry winters [35]. The mean annual temperature is 14.7 °C, and the mean annual precipitation is 583.6 mm, with a trend toward warmer and wetter conditions observed over the past four decades [36]. Vegetation in the reserve consists of warm temperate deciduous broad-leaved forest and coniferous broad-leaved mixed forest, with well-preserved natural secondary forests [35].

### 2.2. Study Subjects

The study subjects were Taihangshan macaques inhabiting the Wangwu area (35°11′56″ N, 112°16′17″ E) and Wulongkou area (35°11′48″ N, 112°41′27″ E) in Jiyuan City, Henan Province, China. A group of Taihangshan macaques (designated as the WW-1 group) in the Wangwu area has been monitored by the forestry rangers of Yugong Forestry Farm since late 2002. Group size [37] and adult males were identified by the rangers before 2007 and by our team members (postgraduate students and JDT) thereafter. Individual identification was based on facial characteristics (e.g., color, speckles, scars) and other traits such as body size and pelage color. Age categories were defined as newborns (0–8 months), juveniles (8 months to 4.5 years for females and to 4.5 years for males), adult males (>4.5 years), and adult females (>4.5 years) [34]. We defined newborns as 0–8 months and adult females as >4.5 years because we conducted annual demographic surveys in late December each year, when the monkeys had generally reached the midpoint between birthdays. Immatures included newborns and juveniles. The mating period covers from September to December, and the birthing period spreads from February to August [35]. Group compositions before and after alpha male replacement events in the studied groups were tabulated in Table 1.

The Wulongkou area has been developed for tourism since the early 1980s. A group of wild Taihangshan macaques (approximately 50 individuals, designated as the original WLK-1 group) was attracted from the mountains using food baits provided by staff of the Administration Bureau of Wulongkou Scenic Spot in Jiyuan. Since then, this group has remained in the area and becomes habituated to tourists. Over the past four decades, numerous new macaque groups have derived from this original group. Some of these newly formed groups have remained in the area (e.g., WLK-1A, WLK-1B, and WLK-3), while others have left and moved to neighboring regions. Additionally, a group of approximately 12 individuals was translocated from a neighboring area (approximately 10 km away to the northwest) to the Wulongkou area in the late 1990s and has resided there since then. Currently, five groups of Taihangshan macaques inhabit the Wulongkou area, four of which (WLK-1A, WLK-1B, WLK-2, and WLK-3) are regularly observed by tourists.

Due to historical human disturbances (e.g., logging and farming) and local environmental constraints such as relatively low annual precipitation, developed hills, and poor soil, food provisioning has been provided by designated staff from the Administration Bureau of Jiyuan Wulongkou Scenic Spot since the inception of macaque-based tourism. Nevertheless, the macaques continue to freely forage for natural food resources [38,39]. Specifically, food provisioning occurs twice daily during the winter period (November to March) and once daily during the summer period (April to October). Supplemental food consists primarily of dried yellow maize, along with dried peanuts, dried wheat seed, seasonal vegetables (e.g., carrot, Chinese cabbage, cucumber, sweet potato, pumpkin), and fruits (e.g., apple, peach, cherry, banana, jujube, watermelon) [38]. After 2018, numerous “warm-hearted” individuals began regularly feeding the macaques with similar food items. Since 2013, our team has conducted individual identification of Taihangshan macaques in this area, during which we intentionally identified alpha males through field observations.

### 2.3. Dominance Hierarchy and Statistical Analyses

To assess the social rank of adult males in the studied rhesus monkey groups, we collected the agonistic behaviors using the ad libitum sampling method [40,41]. Social ranks were estimated using David’s Score method [42], implemented in R version 4.5.3 [43] with the package “EloRating”(version 0.46.18) [44]; detailed procedures are provided in the Supplementary Materials. Natality was calculated as the number of newborns (alive or deceased) relative to the number of adult females. Newborn morality was defined as the percentage of ceased newborns relative to the total number of newborns. The proportion of immatures was calculated as the percentage of immatures relative to the group size. Adult sex ratio was calculated as the ratio of the number of adult females to the number of adult males. A paired *t*-test was used to compare variables before and after alpha male replacement events, with the significance level set at *p* < 0.05. Statistical analyses were conducted by R version 4.5.3 (https://www.R-project.org/), and figures comparing variables before and after alpha male replacement were also generated with R version 4.5.3 without further packages. Descriptive data are presented as mean ± SD.

### 2.4. Ethical Note

The performance of this study adhered to the legal requirements of China and was permitted by the Jiyuan Administration Bureau of the Taihangshan Macaque National Nature Reserve. All research activities reported in this article complied with Chinese legal requirements.

## 3. Results

### 3.1. Timing of Alpha Male Replacement Events

In March of 2013, the alpha male of the WW-1 group changed from HB to YS (Table 1). This group underwent fission beginning in March 2020, ultimately forming two groups (WW-1A and WW-1B) by December 2020. Adult male YS retained the alpha rank in WW-1A, the larger of the two groups, which comprised 34 adult individuals, while the alpha male of the WW-1B group, which comprised 16 adult individuals, was KET, who was born in WW-1 in 2011. In the Wulongkou area, alpha male replacement events were recorded as follows: WLK-1 in March 2017; WLK-1A in December 2019 and December 2020; WLK-1B in December 2018; WLK-2 in January 2020 and October 2024; WLK-3 in October 2021 and January 2025 (Table 1). The most recent Group Fission event in the Wulongkou area occurred in the WLK-1 group in March 2017, resulting in the formation of WLK-1A and WLK-1B (Table 1). Among the alpha male replacement events, three occurred in December, two in October, two in January, and two in March. Additionally, both Group Fission events occurred in March.

### 3.2. Characteristics of Alpha Males

From February 2009 to December 2025, we recorded a total of eleven cases of alpha male replacements in rhesus monkeys inhabiting the Taihangshan Macaque National Natural Reserve (Jiyuan Section), Henan Province, China (Table 2). The average age at which adult males attained the alpha position was 10.2 ± 4.1 years (*n* = 11). The average social rank prior to becoming alpha was 3.91 ± 3.05 (*n* = 11), with the second and third rank accounting for 36.36% and 36.36% of cases, respectively. In contrast, alpha males in newly formed groups had very low social ranks in their original groups (7th and 12th). Furthermore, the average tenure of alpha males in recent years (after 2017) was 2.6 ± 1.4 years (*n* = 5), whereas tenure before 2017 appeared to be longer (>7.5 ± 2.9 yr, *n* = 4).

### 3.3. Potential Factors Influencing Alpha Male Replacement

**Figure 1:**
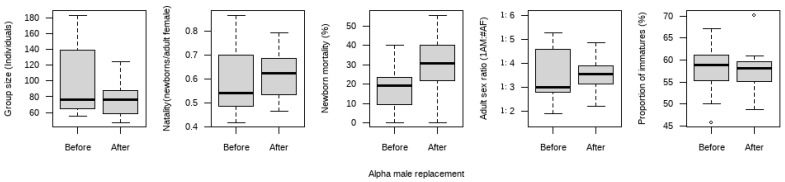
Comparisons of variables between before and after the occurrence of alpha male replacements. AM means adult male, and AF means adult female. The box represents the interquartile range (IQR), the thick line indicates the median, and the whiskers extend to the most extreme data points within (1.5 *IQR). The blank circles denote outliers.

We compared group size, natality, newborn mortality, the ratio of adult males to adult females, and the proportion of immatures before and after alpha male replacement events (Figure 1). No significant differences were found in group size, natality, the ratio of adult males to adult females, or the proportion of immatures (paired *t*-test, all *p* values ≥ 0.11). However, newborn mortality after alpha male replacement events was significantly higher than that before the events (paired *t*-test, t = 3.13, df = 11, *p* = 0.0096).

### 3.4. Characteristics of Different Types of Alpha Male Replacement

**Figure 2:**
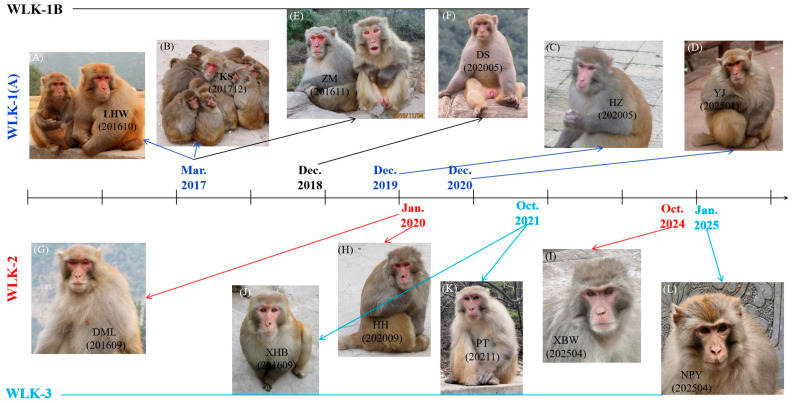
Alpha males of WLK-1A, WLK-1B, WLK-2, and WLK-3 groups. (**A**) shows the alpha male (LHW) of WLK-1 before March 2017; (**B**) shows the alpha male (KS) of WLK-1A from July 2017 to December 2019; (**C**) shows the alpha male (HZ) of WLK-1A from January 2020 to December 2020; (**D**) shows the alpha male (YJ) of WLK-1A after January 2021; (**E**) shows the alpha male (ZM) of WLK-1B from March 2017 to December 2018; (**F**) shows the alpha male (DS) of WLK-1B after January 2019; (**G**) shows the alpha male (DML) of WLK-2 before February 2020; (**H**) shows the alpha male (HH) of WLK-2 from February 2020 to November 2024; (**I**) shows the alpha male (XBW) of WLK-2 after November 2024; (**J**) shows the alpha male (XHB) of WLK-3 before November 2021; (**K**) shows the alpha male (PT) of WLK-3 from November 2021 to January 2025; and (**L**) shows the alpha male (NPY) of WLK-3 after January 2025. The numbers in brackets indicate the dates when the pictures were taken.

In total, we recorded eleven cases of alpha male replacements across the studied rhesus monkey groups (Table 2) and identified three types based on the established definition. Succession was the most common type, accounting for the majority of cases (8/11). Several instances of succession were observed. In March 2017, the alpha male LHW (Figure 2A) left WLK-1 group, which subsequently split into two groups, WLK-1A and WLK-1B, and KS (Figure 2B) assumed the alpha position in WLK-1A. Following the disappearance of KS in January 2020, HZ (Figure 2C) became the alpha male of WLK-1A and was later replaced by YJ (Figure 2D) after HZ’s disappearance in January 2021. In the WLK-1B group, DS (Figure 2F) became alpha male following the disappearance of ZM (Figure 2E) in January 2019. In the WLK-2 group, HH (Figure 2H) became alpha male after the disappearance of the former alpha male DML (Figure 2G) in February 2020 and was subsequently succeeded by XBW (Figure 2I) after his disappearance in November 2024. In the WLK-3 group, PT (Figure 2K) assumed the alpha position in November 2021 following the disappearance of the previous alpha male XHB (Figure 2J). PT was later succeeded by NPY (Figure 2L) after falling ill, experiencing difficulties with feeding and moving, and passing away on 26 January 2025.

**Figure 3:**
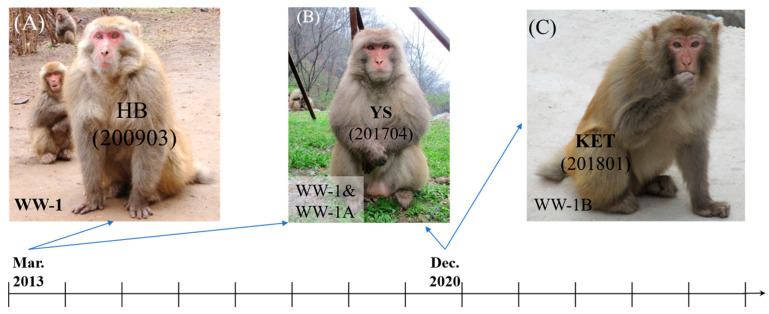
Alpha males of the WW-1, WW-1A, WW-1B, and WLK-1B groups. (**A**) shows the alpha male (HB) of WW-1 before 2013, (**B**) shows the alpha male (YS) of WW-1 after 2013, and (**C**) shows the alpha male (KET) of WLK-1B after July 2017. The numbers in the bracket indicate the dates when the pictures were taken.

Moreover, we documented one case of Rank Reversal and two cases of Group Fission. In March 2013, the alpha male HB (Figure 3A) of the WW-1 group was replaced by a subordinate co-resident male, YS (Figure 3B) through ritualized aggression (e.g., displacing and threatening) rather than combative aggression (e.g., chasing, seizing, and biting). HB remained in the WW-1 group until September 2017, and given his estimated advanced age (approximately 33 years of age), he likely passed away. The WW-1 group underwent division into several small groups between March 2020 and November 2020, ultimately forming two groups, WW-1A and WW-1B, by December 2020. YS retained the alpha position in the WW-1A group, which inherited the key adult males and females and maintained a larger group size. KET, born in WW-1 in 2011, became the alpha male of WW-1B in December 2020 (Figure 3C). In the Wulongkou area, the WLK-1 group, the largest group in Jiyuan Wulongkou area at that time, split into several smaller groups in March 2017, eventually forming WLK-1A and WLK-1B by July 2017. Prior to the fission, LHW (Figure 2A) served as the alpha male of the WLK-1 group; he subsequently left the group with several adult females and juveniles, moved to a nearby area, and disappeared after April 2017. The newly formed WLK-1A group inherited the key members of the original WLK-1 group, including the beta- and third-ranking males, as well as most adult females and their offspring, totaling 105 individuals. In contrast, WLK-1B consisted of 47 individuals and shifted its home range from the core patrol area of WLK-1 to the periphery of the Jiyuan Wulongkou area. Its alpha male, ZM (Figure 2E), had previously been a subordinate co-resident male in the WLK-1 group.

## 4. Discussion

Alpha male replacement commonly occurs in non-human primate species with multi-male and multi-female societies, yet information on its characteristics remains limited for most species, primarily due to the long lifespan of individuals and the unpredictable nature of such events [5]. In this study, we present eleven cases of alpha male replacement in rhesus monkeys, providing a general picture of their characteristics.

Being an alpha male confers priority access to resources in multi-male and multi-female primate societies, yet attaining the alpha position requires both strategy and tactics [5]. Rhesus monkeys are seasonal breeders, with mating and birthing seasons primarily occurring in autumn and early winter (September to December) and spring and early summer (March to June), respectively [45,46]. In the present study, alpha male replacements occurred during the mating season (5/11), gestation period (2/11), and early birthing season (2/11), while both Group Fission events began in the early birthing season (March). This pattern may be linked to the pursuit of mating opportunities. During the mating season, adult males, whether extra-group or within group individuals, may attempt to obtain or maximize their opportunities to fertilize females, who tend to conceive during their first mating cycle [45]. Consequently, competition for reproductive access during this period may lead adult males to challenge the established dominance hierarchy, potentially triggering alpha male replacement [31]. One reported case of alpha male replacement in Cayo Santiago rhesus monkeys occurred at the start of the mating season (late February 2013) [8]. A predominance of alpha male replacements during the mating season has also been reported in another seasonal breeder, Formosan macaques [21], but was not observed in the non-seasonal breeder crested macaque [7,47]. Some females that fail to conceive during the main mating season may seek mating opportunities later (e.g., in January) [45,48], which could also increase the risk of alpha male replacement. Additionally, we recorded two cases of Group Fission occurring in March, at the onset of the birthing season [46], consistent with findings in rhesus monkeys in Northern India [49]. The timing may be attributed to reduced social cohesion and a return to optimal group size. For instance, rhesus monkeys in Northern India tend to undergo fission when group size exceeds the optimum (e.g., approximately 120 individuals), often doing so prior to the birthing season [49]. In such cases, group cohesiveness deteriorates before fission, with females exhibiting more spatially loose associations [50].

The types of alpha male replacement are commonly classified as Takeover, Rank Reversal, Succession, Waltz-in, and Group Fission, all of which have been observed across multi-male and multi-female primate species, and a given species may exhibit one or more of these types [5]. In the present study, rhesus monkeys displayed three types of alpha male replacement including Succession, Fission and Rank Reversal, with Succession being the most frequent (8/11), consistent with our prediction. Rhesus monkeys live in multi-male and multi-female societies characterized by female philopatry and male dispersal around sexual maturity [29]. The predominance of Succession in our study populations aligns with the inference of previous studies [5,8]. The pattern may be explained by the finding that the social rank of adult males in this species is more closely associated with tenure than with fighting ability [51]. Support for this interpretation comes from the observed Rank Reversal event, in which the natal male YS replaced the previous alpha male HB, who was estimated to be very old (approximately 29 years of age) at the time of replacement in 2013. The long-term serving period of HB as alpha male in the WW-1 group may be attributed to the presence of only one group in the area and the limited number of adult males prior to 2013 [37]. In contrast, crested macaques, classified as Grade 4 (extremely tolerant) in social style, exhibited Takeover (5/9) and Succession (4/9), showing no clear bias toward a particular type, unlike rhesus monkeys (Grade 1) [30]. However, further data are needed to test the influence of social style on the type of alpha male replacement in macaques.

Group Fission may also be common in rhesus monkeys [49]. Group size typically increases with the accumulation of adult females and their offspring. However, due to resource limitations in the wild, group size cannot grow indefinitely; thus, Group Fission may serve to maintain an optimal group size [49,50]. When a new group forms from a larger one, the alpha male of the new group typically originates from the original group or immigrates from outside (i.e., Waltz-in), although the later was not observed in this study. Additionally, Takeover, though rare, has been reported in Cayo Santiago rhesus monkeys [8], but no such evidence was found in the present rhesus populations during the study period (February 2009 to April 2025). This may be attributed to the rigid dominance hierarchy within rhesus groups, particularly among adult males, which likely increases the difficulty for extra-group adult males to immigrate and attain the alpha position [5].

Becoming an alpha male in many social-living non-human primates is thought to confer significant advantages, including priority access to limited resources such as food resources and reproductive females [2]. However, a complex and often interactive set of factors determine whether a male successfully attains alpha rank [52,53]. In the present study, the average age at which males became alpha was 10.3 years, suggesting that males typically reach full body size before acquiring the alpha position. Although sexual maturity is generally defined as older than six years of age, full body size in adult males may be attained later [33,45]. This pattern may also be linked to the predominance of Succession as the primary type of alpha male replacement. A previous study has demonstrated that social rank among adult male rhesus monkeys tends to correlate with tenure [45], indicating that aging and full body size may increase not only the likelihood of achieving a higher social rank but also the opportunity to become an alpha male.

The average social rank of adult males prior to becoming alpha was 3.91 (±3.05), suggesting that beta- or third-ranking males have a higher probability of ascending to the alpha position. In rhesus monkey society, which is characterized by a strict linear dominance hierarchy, beta- or third-ranking adult males typically suppress all other males except the alpha or beta individuals. Consequently, they are well positioned to assume the alpha position upon the disappearance or death of the sitting alpha male. However, the tenure of alpha males may be influenced by individual health conditions and other factors. Research has shown that higher-ranking individuals may be more susceptible to chronic stress [54], suggesting that alpha males may experience accelerated health deterioration. This is supported by the case of PT (born in 2013), the alpha male of WLK-3, who was observed with diarrhea in early January 2025 and passed away on 26 January 2025.

Furthermore, the tenure of recent alpha males (mean ± SD: 2.7 ± 1.4 years) appeared shorter than that of alpha males before 2017 (>7.5 years), a difference that may be attributable to changes in human disturbance. Prior to 2018, regular food provisioning was conducted principally by designated staff of the Administration Bureau of Jiyuan Wulongkou Scenic Spot, with feeding times restricted to 17:00–18:00 year-round and an additional session from 10:00 to 11:00 during the winter period. The narrow time window allowed most group members to gather and share the supplemented food, which may have reinforced the dominance hierarchy and indirectly strengthened social cohesion within the rhesus group. Under such conditions, alpha males were likely able to maintain their positions for longer periods, relying primarily on physical intimidation, coercive power, skills, and intelligence [5,52,53]. After 2018, however, food provisioning was no longer limited to staff but was also carried out by numerous “warm-hearted” individuals who provided food continuously throughout the day at both fixed and scattered locations. This shift could likely reduce social cohesion by lowering the spatial and temporal aggregation of rhesus group members, thereby indirectly destabilizing the dominance hierarchy. Consequently, alpha male tenure has tended to be shorter in recent years under these altered conditions.

Alpha male replacement can be influenced by factors such as group size, adult sex ratio, and the proportion of immatures within a rhesus group, and may in turn affect natality and newborn mortality [5]. In the present study, we found that newborn mortality tended to be higher following alpha male replacement events, whereas no significant differences were observed for the other variables examined. The finding aligns with studies on alpha male replacement in white-throated capuchins (*Cebus capucinus imitator*) [55], male immigration in Angolan colobus (*Colobus angolensis ruwenzorii*) [56], and male replacement in gibbons [19]. However, the underlying mechanisms appear to differ between rhesus monkeys and these other primate species. In the later species, elevated newborn mortality is largely attributed to infanticide and, in some cases, protection from related males [19,55,56]. In contrast, we observed no cases of infanticide in our studied rhesus groups, although infanticide has been reported in this species elsewhere [57]. Rhesus monkeys live in multi-male and multi-female groups with a promiscuous mating system, in which peripheral adult males may also have opportunities to sire offspring [8,29]. Theoretically, the costs of infanticide would be prohibitively high for both resident and extra-group males under such conditions. A more plausible explanation for the observed increase in newborn mortality following alpha male replacement may be heightened social anxiety and reduced protection from mothers and kin resulting from social instability triggered by the replacement event [8,56].

## 5. Conclusions

Rhesus monkeys are the most widely distributed non-human primate species and serve as one of the most well-studied model organisms in primatology. Their dominance hierarchy and the role of the alpha male have been comprehensively investigated [33,54,58]. However, detailed information on the characteristics of alpha males and the dynamics of alpha male replacement remains limited [5]. In this study, we documented eleven cases of alpha male replacement and provided a description of alpha male characteristics in rhesus monkeys. The key finding is that Succession constituted the predominant type of alpha male replacement, a pattern consistent with the species’ extremely despotic social style. Given the limitations of sample size and the human-influenced nature of the study area, we recommend that additional evidence from other locations would help deepen our understanding of the processes underlying alpha male attainment and tenure in rhesus monkeys, as well as facilitate further testing of the relationship between social style and the type of alpha male replacement.

## Figures and Tables

**Table 1:** Group compositions before and after alpha male replacement in the studied groups of Taihangshan macaques.

Group ID	Date For Counting Group Size	Time Period	Group Sizes	Number of Adult Males	Number of Adult Females	Number of Juveniles	Number of All/Dead Newborn	Adult Sex Ratio	Natality	Newborn Mortality	Proportion of Immatures
WW-1	2012.12	Before	59	7	19	22	11/0	2.71	57.895	0.000	0.559
WW-1	2013.12	After	77	7	24	27	19/0	3.43	79.167	0.000	0.597
WW-1	2019.12	Before	139	14	40	60	28/3	2.86	70.000	10.714	0.612
WW-1A	2020.12	After	124	10	40	55	24/5	4.00	60.000	20.833	0.597
WW-1B	2020.12	After	47	5	11	28	8/3	2.20	72.727	37.500	0.702
WLK-1	2016.12	Before	183	12	59	87	32/7	4.92	54.237	21.875	0.612
WLK-1A	2017.12	After	105	7	34	46	24/6	4.86	70.588	25.000	0.610
WLK-1A	2018.12	Before	113	8	42	51	18/6	5.25	42.857	33.333	0.558
WLK-1A	2019.12	After	89	9	31	39	20/10	3.44	64.516	50.000	0.551
WLK-1A	2020.12	Before	81	10	34	22	18/3	3.40	52.941	16.667	0.457
WLK-1A	2021.12	After	86	8	36	29	19/6	4.50	52.778	31.579	0.488
WLK-1B	2017.12	After	47	4	15	24	7/3	3.75	46.667	42.857	0.596
WLK-1B	2018.12	Before	55	4	17	28	8/2	4.25	47.059	25.000	0.618
WLK-1B	2019.12	After	57	5	18	30	9/5	3.60	50.000	55.556	0.596
WLK-2	2019.12	Before	71	8	24	33	10/4	3.00	41.667	40.000	0.549
WLK-2	2020.12	After	75	10	26	30	14/5	2.60	53.846	35.714	0.520
WLK-2	2023.12	Before	72	11	25	17	20/1	2.27	80.000	5.000	0.500
WLK-2	2024.12	After	76	7	26	31	17/5	3.71	65.385	29.412	0.566
WLK-3	2020.12	Before	55	6	18	24	9/2	3.00	50.000	22.222	0.564
WLK-3	2021.12	After	60	7	20	23	11/1	2.86	55.000	9.091	0.550
WLK-3	2024.12	Before	70	8	15	35	13/1	1.88	86.667	7.692	0.671
WLK-3	2025.12	After	79	8	27	30	18/4	3.38	66.667	22.222	0.557

Note: Time period means the period before or after the occurrence of alpha male replacement. Adult sex ratio means the ratio of the number of adult females to the number of adult males.

**Table 2:** Summary information on the alpha males in each group of Taihangshan macaques.

Group ID	Alpha Male ID	Estimated Birth Year	Age at Becoming Alpha Male	Age at Losing Alpha Rank	Social Rank Before Becoming Alpha Male	Time Period for Alpha Male	Tenure for Alpha Male
WW-1	HB	~1984	NA	~29	NA	2002<–2013.03	>11 yr
	YS	2006	7	NA	3rd	2013.04–>2020.03	6 yr
WW-1A	YS	2006	7	NA	1rd	2020.04–2025.12	5 yr 8 mon
WW-1B	KET	2011	9.5	NA	7th	2020.11–NA	NA
WLK-1	LHW	NA	NA	NA	NA	2013.03<–2017.03	>4 yr
WLK-1A	KS	~1997	~20	~22	2nd	2017.04–2019.12	2 yr 8 mon
	HZ	2011	9	10	3rd	2020.01–2020.12	1 yr
	YJ	2013	8	NA	2nd	2021.01–2025.12	>5 yr
WLK-1B	ZM	2010	7	9	12th	2017.04–2018.12	1 yr 8 mon
	DS	2012	7	NA	3rd	2019.01–2025.12	>6 yr 7 mon
WLK-2	DML	NA	NA	NA	NA	2013<–2020.01	>7 yr
	HH	2005	15	20	4th	2020.02–2024.10	4 yr 8 mon
	XBW	2012	12	NA	2nd	2024.11–2025.12	>1 yr 1 mon
WLK-3	XHB	~2003	NA	~18	NA	2013<–2021.10	>8 yr
	PT	2013	8	11	3rd	2021.11–2025.01	3 yr 1 mon
	NPY	2015	10	NA	2nd	2025.02–2025.12	>10 mon

Note: NA means no available data for the item. The social rank before becoming alpha male was estimated as the social rank among adult males in the rhesus monkey group. yr means year. ~ means approximately. yr means year(s). mon means month(s).

## Data Availability

The data analyzed in this study are available from the corresponding author upon reasonable request.

## Supplementary Materials

The following supporting information can be downloaded at: https://www.mdpi.com/article/10.3390/ani16101495/s1, Table S1: Dominance hierarchy among adult males in WW-1 during 2012.03 and 2012.12; Table S2: Dominance hierarchy among adult males in WW-1 during 2019.03 and 2020.02; Table S3: Dominance hierarchy among adult males in WLK-1 during 2016.07 and 2017.03; Table S4: Dominance hierarchy among adult males in WLK-1A during 2019.07 and 2019.12; Table S5: Dominance hierarchy among adult males in WLK-1A during 2020.07 and 2020.12; Table S6: Dominance hierarchy among adult males in WLK-1B during 2019.07 and 2019.12; Table S7: Dominance hierarchy among adult males in WLK-2 during 2019.07 and 2019.12; Table S8: Dominance hierarchy among adult males in WLK-2 during 2024.07 and 2024.12; Table S9: Dominance hierarchy among adult males in WLK-3 during 2021.07 and 2021.10; Table S10: Dominance hierarchy among adult males in WLK-3 during 2024.07 and 2024.12. Refs. [59,60] are cited in Supplementary Materials.

## Abbreviations

The following abbreviations are used in this manuscript:

spp.	species pluralis
OMU	one-male unit
AMU	all-male unit
mm	millimeter
mon	month
kg	kilogram
km	kilometer
m	meter
N	northern latitude
E	eastern longitude
AM	adult male
AF	adult female
i.e.	id est or that is to say
SD	standard deviation
ID	identification code
yr	year
NA	no available data
IQR	interquartile range
