# Supplementary material for: Succession Dominates Alpha Male Replacement in Despotic Rhesus Monkeys: Insights from a Long-Term Study in the Taihang Mountains, Henan Province, China
Source: Animals (Basel). 2026 May 13;16(10):1495. doi: 10.3390/ani16101495 (PMC13203206; doi:10.3390/ani16101495)
Supplement: Supplementary file 1 [file animals-16-01495-s001.zip › animals-4247038-supplementary.pdf]

animals

MDPI

Supplementary file to article:

Succession Dominates Alpha Male Replacement in Despotic Rhesus Monkeys: Insights from a Long-Term Study in the Taihang Mountains, Henan Province, China

Haotian Xu [1,2,†], Bo Zhi [1,2,†], Longhui Hu [1,2], Jundong Tian [1,2,*] and Jiqi Lu [1,2]

[1] School of Life Sciences, Zhengzhou University, Zhengzhou 450001, China; 16638114660@163.com (H.X.); 18568637709@163.com (B.Z.); 15890956623@163.com (L.H.)
[2] Institute of Biodiversity and Ecology, Zhengzhou University, Zhengzhou 450001, China
* Correspondence: tianjd@zzu.edu.cn
† These authors contributed equally to this work.

The assessment of social ranks among adult males during the study period before the occurrence of alpha male replacement

Methods

Behavioral data collection

To assess the social rank of adult males in the studied rhesus monkey groups, we collected the agonistic behaviors via ad libitum sampling method (Altman 1974; Bateson and Martin 2021). We have trained the postgraduate students for daily behavioral data collection, such as individual identification, agonistic behavior and daily activity time budget, via senior students training junior students. Each of those postgraduate students have been in the field for conducting their project for at least 15 months, normally from July to the following September. During the overlap period (from July to September), the senior postgraduate students would train the junior postgraduate students, and the supervisors (JDT & JQL) would assess the efficiency before starting collect formal behavioral data. However, we did not quantitatively assess the inter-reliability of the students' sampling efforts, and therefore acknowledge that undetected variations may exist. This two-step procedure could not only continuously accumulate fundamental data but also minimize the bias in data collections performed by different postgraduate students.

The collecting periods of the agonistic data for different rhesus monkey groups varied according to the dates of the alpha male replacements, which were sub-datasets from the above-mentioned daily behavioral dataset. In specific, the datasets of agonistic data (e.g., replace, threaten, chase, seize, bite; Tian et al., 2011) have been collected from March 2012 to December 2012 and March 2019 to March 2020 for WW-1, from July 2016 to March 2017 for WLK-1, from July 2019 to December 2019 and from July 2020 to December 2020 for WLK-1A, from July 2018 to December 2018 for WLK-1B, from July 2019 to December 2019 and from July 2024 to December 2024 for WLK-2, from July 2021 to October 2021 and July 2024 to December 2024 for WLK-3. The postgraduate students have collected agonistic data for two weeks each month, and the time period for collecting agonistic data ranged from 08:00 to approximately 17:00 during the winter period (November to March) and approximately 18:00 during the summer period (April to October). The initiators and receivers involved in the agonistic events were recorded, and the winners and losers were also recorded in order to estimate their social rank. In specific, the agonistic behavior of Replace was defined as that one monkey occupied the location where the other one took without obvious conflicts; Threaten was defined as one monkey stared the other one

often accompanied with vocalizations and facial expressions often without physical contact; Chase was defined as pursuit past the point at which a victim began to flee, and could include physical contact; Seize was defined as that a monkey tried to catch the other one with its hands but often did not bite; Bite, the most intense type, was defined as that a monkey bited another one with its mouth (Judge & de Waal, 1997; Tian et al., 2011).

Data analysis

We applied David's Score method (Gammell et al. 2003) to estimate the social rank of adult males of each group. In specific, we performed the statistics with R version 4.5.3 (R Core Team 2026) and the package "EloRating" (Neumann and Kulik 2024) to calculate the David's Score of the adult males in each group before the occurrence of alpha male replacements.

Results

Dominance hierarchy among adult males in WW-1

The social ranks among adult males in WW-1, from highest to lowest, were HB > ZM > YS > XBL > PZ > XQ during 2012.03 and 2012.12 (Table S1) and YS > XBL > CX > ZM > XL > GL > KET > JE > HZ > BZM > BB > BE > LZ > HYD (Table S2).

Table S1. Dominance hierarchy among adult males in WW-1 during 2012.03 and 2012.12

		Losers							David's Score	
		HB	ZM	YS	XBL	PZ	XQ	BT	DS	normDS
Winners	HB*	0	18	17	9	7	5	3	21.00	6.00
	ZM	0	0	2	27	25	8	5	11.98	4.71
	YS#	0	0	0	22	20	11	13	5.17	3.74
	XBL	0	9	6	0	1	15	9	3.25	3.46
	PZ	0	1	1	0	0	6	7	-6.40	2.09
	XQ	0	0	0	0	0	0	5	-14.00	1.00
	BT	0	0	0	0	0	0	0	-21.00	0.00

Note: * means the alpha male, # means the following alpha male of the same group, DS means the calculated values of David's Score, normDS means the normalized values of David's Score, Two or three capitalized letters means the ID of adult males.

Table S2. Dominance hierarchy among adult males in WW-1 during 2019.03 and 2020.02

		Losers														David's Score	
		YS	XBL	CX	ZM	XL	GL	KET	JE	HZ	BZM	BB	BE	LZ	HYD	DS	normDS
Winners	YS*,#	0	17	0	15	19	9	8	10	0	5	5	4	4	8	51.00	10.14
	XBL	0	0	16	0	25	22	18	21	16	19	13	16	11	13	46.00	9.79
	CX	0	0	0	0	20	24	0	23	17	10	13	9	0	7	30.00	8.64
	ZM	0	0	0	0	27	0	21	0	0	15	0	0	13	11	17.50	7.75
	XL	0	0	0	0	0	17	0	0	13	9	11	7	0	7	9.00	7.14
	GL	0	0	0	0	0	0	19	0	12	0	0	0	7	9	2.00	6.64
	KET†	0	0	0	0	0	0	0	11	0	9	5	0	0	0	1.00	6.57
	JE	0	0	0	0	0	0	0	0	5	0	7	9	0	0	-4.50	6.18
	HZ	0	0	0	0	0	0	0	5	0	9	7	11	0	9	-9.50	5.82
	BZM	0	0	0	0	9	0	0	0	9	0	0	5	9	8	-11.00	5.71
	BB	0	0	0	0	0	0	0	0	0	0	0	0	11	13	-23.50	4.82
	BE	0	0	0	0	0	0	0	0	0	5	0	0	7	1	-25.00	4.71
	LZ	0	0	0	0	0	0	0	0	0	0	0	7	0	9	-33.00	4.14

https://doi.org/10.3390/ani16101495

HYD	0	0	0	0	0	0	0	0	0	0	0	0	9	0	-50.00	2.93

Note: *,# means the male kept the alpha position before and after group fission, † means the alpha male for a newly-formed group after group fission, DS means the calculated values of David's Score, normDS means the normalized values of David's Score, Two or three capitalized letters means the ID of adult males.

Dominance hierarchy among adult males in WLK-1

The social ranks among adult males in WLK-1, from highest to lowest, were LHW > KS > HB > LS > CZ > ML > ZF > HZ > JB > DS > XZ > ZM during 2016.07 and 2017.03 (Table S3).

Table S3. Dominance hierarchy among adult males in WLK-1 during 2016.07 and 2017.03

		Losers												David's Score	
		LHW	KS	HB	LS	CZ	ML	ZF	HZ	JB	DS	XZ	ZM	DS	normDS
Winners	LHW*	0	7	12	6	6	5	3	1	0	0	3	2	50.20	9.68
	KS#	0	0	19	23	17	15	17	10	9	0	7	3	43.20	9.10
	HB	0	0	0	17	0	25	19	13	11	15	10	7	26.00	7.67
	LS	0	0	0	0	13	15	11	6	0	10	8	5	19.00	7.08
	CZ	0	0	0	0	0	17	9	12	13	7	10	9	14.00	6.67
	ML	0	0	0	0	0	0	7	3	0	0	0	3	-4.00	5.17
	ZF	0	0	0	0	0	0	0	13	1	0	1	1	-9.80	4.68
	HZ	0	0	0	0	0	0	0	0	1	1	1	0	-20.00	3.83
	JB	0	0	0	0	0	0	0	0	0	1	1	1	-20.00	3.83
	DS	0	0	0	0	0	0	0	0	0	0	8	0	-26.00	3.33
	XZ	0	0	0	0	0	0	0	0	0	2	0	0	-39.60	2.20
	ZM	0	0	0	0	0	0	0	0	0	0	0	0	-33.00	2.75

Note: * means the alpha male, # means the following alpha male of the same group, DS means the calculated values of David's Score, normDS means the normalized values of David's Score, Two or three capitalized letters means the ID of adult males.

Dominance hierarchy among adult males in WLK-1A

The social ranks among adult males in WLK-1A, from highest to lowest, were KS > HB > HZ > LS > CZ > ML > ZF > YL > YJ during 2019.07 and 2019.12 (Table S4) and HZ > YJ > LS > ML > BL > DB > DC > XC > XH > ZF during 2020.07 and 2020.12 (Table S5).

Table S4. Dominance hierarchy among adult males in WLK-1A during 2019.07 and 2019.12

		Losers									David's Score	
		KS	HB	HZ	LS	CZ	ML	ZF	YL	YJ	DS	normDS
Winners	KS*	0	7	11	5	8	3	2	1	3	33.88	7.76
	HB	1	0	13	17	11	9	10	12	7	27.13	7.01
	HZ#	0	0	0	12	13	7	9	5	5	17.00	5.89
	LS	0	0	0	0	9	3	3	5	2	8.00	4.89
	CZ	0	0	0	0	0	11	7	0	6	-2.00	3.78
	ML	0	0	0	0	0	0	8	7	3	-9.00	3.00
	ZF	0	0	0	0	0	0	0	10	4	-18.00	2.00
	YL	0	0	0	0	0	0	0	0	7	-22.00	1.56
	YJ	0	0	0	0	0	0	0	0	0	-35.00	0.11

Note: * means the alpha male, # means the following alpha male of the same group, DS means the calculated values of David's Score, normDS means the normalized values of David's Score, Two or three capitalized letters means the ID of adult males.

Table S5. Dominance hierarchy among adult males in WLK-1A during 2020.07 and 2020.12

		Losers										David's Score	
		HZ	LS	ML	YJ	BL	DB	DC	XC	XH	ZF	DS	normDS
Winners	HZ*	0	9	5	7	11	5	0	5	2	2	37.00	8.20
	LS	0	0	11	0	5	8	7	9	5	0	22.00	6.70
	ML	0	0	0	0	7	5	6	0	7	9	11.00	5.60
	YJ#	0	7	10	0	6	0	9	5	11	3	27.00	7.20
	BL	0	0	0	0	0	11	8	10	0	9	3.00	4.80
	DB	0	0	0	0	0	0	7	6	7	3	-3.00	4.20
	DC	0	0	0	0	0	0	0	10	5	7	-13.00	3.20
	XC	0	0	0	0	0	0	0	0	7	8	-19.00	2.60
	XH	0	0	0	0	0	0	0	0	0	3	-27.00	1.80
	ZF	0	0	0	0	0	0	0	0	0	0	-38.00	0.70

Note: * means the alpha male, # means the following alpha male of the same group, DS means the calculated values of David's Score, normDS means the normalized values of David's Score, Two or three capitalized letters means the ID of adult males.

Dominance hierarchy among adult males in WLK-1B

The social ranks among adult males in WLK-1B, from highest to lowest, were ZM> XZ > DS > HL during 2019.07 and 20119.12 (Table S6).

Table S6. Dominance hierarchy among adult males in WLK-1B during 2019.07 and 2019.12

		Losers				David's Score	
		ZM	DS	XZ	HL	DS	normDS
Winners	ZM*	0	11	15	12	6.00	3.00
	DS#	0	0	1	9	-1.71	1.07
	XZ	0	13	0	14	1.71	1.93
	HL	0	0	0	0	-6.00	0.00

Note: * means the alpha male, # means the following alpha male of the same group, DS means the calculated values of David's Score, normDS means the normalized values of David's Score, Two or three capitalized letters means the ID of adult males.

Dominance hierarchy among adult males in WLK-2

The social ranks among adult males in WLK-2, from highest to lowest, were DML > CM > ELZ > HH > DZ > TM > XML > YDLY during 2019.07 and 2019.12 (Table S7) and HH > SG > ZDLY > YDLY > XB > DZ > EBA during 2020.07 and 2020.12 (Table S8).

Table S7. Dominance hierarchy among adult males in WLK-2 during 2019.07 and 2019.12

		Losers								David's Score	
		DML	HH	CM	ELZ	DZ	TM	XML	YDLY	DS	normDS
Winners	DML*	0	6	10	15	8	7	9	10	28.00	7.00
	HH#	0	0	1	2	10	5	7	8	7.00	4.38
	CM	0	7	0	9	10	6	3	4	18.20	5.78
	ELZ	0	6	1	0	8	9	7	7	10.80	4.85
	DZ	0	0	0	0	0	6	5	2	-4.00	3.00

	TM	0	0	0	0	0	0	7	3	-12.00	2.00
	XML	0	0	0	0	0	0	0	5	-20.00	1.00
	YDLY	0	0	0	0	0	0	0	0	-28.00	0.00

Note: * means the alpha male, # means the following alpha male of the same group, DS means the calculated values of David's Score, normDS means the normalized values of David's Score, Two or three capitalized letters means the ID of adult males.

Table S8. Dominance hierarchy among adult males in WLK-2 during 2024.07 and 2024.12

		Losers							David's Score	
		HH	XBW	ZDLY	YDLY	XB	DZ	EBA	DS	normDS
Winners	HH*	0	11	9	9	8	3	2	19.50	5.79
	XBW#	3	0	15	12	13	9	6	15.50	5.21
	ZDLY	0	0	0	7	9	6	3	7.00	4.00
	YDLY	0	0	0	0	8	5	7	0.00	3.00
	XB	0	0	0	0	0	5	6	-7.00	2.00
	DZ	0	0	0	0	0	0	3	-14.00	1.00
	EBA	0	0	0	0	0	0	0	-21.00	0.00

Note: * means the alpha male, # means the following alpha male of the same group, DS means the calculated values of David's Score, normDS means the normalized values of David's Score, Two or three capitalized letters means the ID of adult males.

Dominance hierarchy among adult males in WLK-3

The social ranks among adult males in WLK-3, from highest to lowest, were XHB > SL > PT > BL > CTB > SBA > SJK during 2021.07 and 2021.10 (Table S9) and PT > BL > SL > CTB > SBA > SJK > XHL > KG during 2024.07 and 2024.12 (Table S10).

Table S9. Dominance hierarchy among adult males in WLK-3 during 2021.07 and 2021.10

		Losers							David's Score	
		XHB	PT	SL	BL	CTB	SBA	SJK	DS	normDS
Winners	XHB*	0	6	7	4	5	3	2	21.00	6.00
	PT#	0	0	1	6	3	5	4	8.17	4.17
	SL	0	5	0	4	5	1	3	12.83	4.83
	BL	0	0	0	0	7	4	2	0.00	3.00
	CTB	0	0	0	0	0	3	4	-7.00	2.00
	SBA	0	0	0	0	0	0	5	-14.00	1.00
	SJK	0	0	0	0	0	0	0	-21.00	0.00

Note: * means the alpha male, # means the following alpha male of the same group, DS means the calculated values of David's Score, normDS means the normalized values of David's Score, Two or three capitalized letters means the ID of adult males.

Table S10. Dominance hierarchy among adult males in WLK-3 during 2024.07 and 2024.12

		Losers								David's Score	
		PT	NPY	SL	CTB	SBA	SJK	XHL	KG	DS	normDS
Winners	PT*	0	13	9	8	5	0	7	4	23.93	6.49
	NPY#	2	0	20	25	23	16	12	15	20.55	6.07
	SL	0	1	0	9	11	5	3	6	12.38	5.05
	CTB	0	0	0	0	6	8	10	3	4.00	4.00
	SBA	0	0	0	0	0	9	4	7	-4.00	3.00

https://doi.org/10.3390/ani16101495

SJK	0	0	0	0	0	0	5	8	-10.87	2.14
XHL	0	0	0	0	0	0	0	4	-19.00	1.13
KG	0	0	0	0	0	0	0	0	-27.00	0.13

Note: * means the alpha male, # means the following alpha male of the same group, DS means the calculated values of David's Score, normDS means the normalized values of David's Score, Two or three capitalized letters means the ID of adult males.

References

1. Altmann J (1974) Observational study of behavior: Sampling methods. Behaviour 49(3/4):227–267. https://doi.org/10.1163/156853974x00534
2. Bateson M, Martin P (2021) Measuring Behaviour: An Introductory Guide (4th edition). Cambridge University Press, Cambridge. https://doi.org/10.1017/9781108776462
3. Gammell MP, de Vries H, Jennings DJ, Carlin CM, Hayden TJ (2003) David⊛s score: A more appropriate dominance ranking method than Clutton-Brock et al.⊛s index. Anim Behav 66(3):601–605. https://doi.org/10.1006/anbe.2003.2226
4. Judge PG, de Waal FBM (1997). Rhesus monkey behaviour under diverse population densities: Coping with long-term crowding. Anim Behav 54:643-662. https://doi.org/10.1006/anbe.1997.0469
5. Neumann C, Kulik L (2024). EloRating: Animal Dominance Hierarchies by Elo Rating. https://doi.org/10.32614/CRAN.package.EloRating.
6. R Core Team (2026) R: A Language and Environment for Statistical Computing. R Foundation for Statistical Computing, Vienna, Austria. https://www.R-project.org/
7. Tian JD, Wang ZL, Lu JQ, Guo XB, Liu JD. 2011. PAE coding system-based ethogram of Taihangshan macaque (Macaca mulatta tcheliensis), Jiyuan, Henan Province, China. Acta Theriologica Sinica, 31(2): 125-140.

Disclaimer/Publisher's Note: The statements, opinions and data contained in all publications are solely those of the individual author(s) and contributor(s) and not of MDPI and/or the editor(s). MDPI and/or the editor(s) disclaim responsibility for any injury to people or property resulting from any ideas, methods, instructions or products referred to in the content.
